# In Situ Programming of CAR-T Cells: A Pressing Need in Modern Immunotherapy

**DOI:** 10.1007/s00005-023-00683-y

**Published:** 2023-07-07

**Authors:** Marta Śledź, Alicja Wojciechowska, Radosław Zagożdżon, Beata Kaleta

**Affiliations:** Department of Clinical Immunology, Medical University of Warsaw, Warsaw, Poland

**Keywords:** Adoptive cellular therapy, CAR-T cells, *In-situ* generation, Gene-editing tools

## Abstract

Chimeric antigen receptor-T (CAR-T) cell-based therapy has become a successful option for treatment of numerous hematological malignancies, but also raises hope in a range of non-malignant diseases. However, in a traditional approach, generation of CAR-T cells is associated with the separation of patient’s lymphocytes, their in vitro modification, and expansion and infusion back into patient’s bloodstream. This classical protocol is complex, time-consuming, and expensive. Those problems could be solved by successful protocols to produce CAR-T cells, but also CAR-natural killer cells or CAR macrophages, in situ, using viral platforms or non-viral delivery systems. Moreover, it was demonstrated that in situ CAR-T induction may be associated with reduced risk of the most common toxicities associated with CAR-T therapy, such as cytokine release syndrome, immune effector cell-associated neurotoxicity syndrome, and “on-target, off-tumor” toxicity. This review aims to summarize the current state-of-the-art and future perspectives for the in situ-produced CAR-T cells. Indeed, preclinical work in this area, including animal studies, raises hope for prospective translational development and validation in practical medicine of strategies for in situ generation of CAR-bearing immune effector cells.

## Introduction

Modern immunotherapies have provided numerous breakthroughs in the current medicine (Jain et al. 2022; Marhelava et al. 2019; Roy et al. 2022). One of the prime examples of this fact is the adoptive cellular therapy, including strategies utilizing chimeric antigen receptors (CAR). Indeed, the CAR-based therapies have recently become a valuable therapeutic option, primarily in hematological malignancies, due to their high remission rates and long-term response to treatment (Yan et al. 2023). However, CAR-based therapies are also expected to revolutionize other areas of medicine because of their potential to act as highly programmable “cellular scalpels”, capable of removing any kind of “unwanted” cells from the organism in a variety of diseases.

CARs are engineered synthetic receptors which have ability to recognize, target, and eliminate cells with specific surface antigens (Sterner and Sterner 2021). Various immune cells have been modified by CARs, including T cells (Byun 2023), natural killer (NK) cells (Oei et al. 2018), as well as macrophages (M) (Sloas et al. 2021). However, CAR-NK and CAR-M cells have been only recently studied and translated into clinical trials (Jogalekar et al. 2022; Pan et al. 2022; Sloas et al. 2021).

There are currently six CAR T-cell therapies approved by the Food and Drug Administration and European Medicines Agency: tisagenlecleucel (Kymriah®), axicabtagene ciloleucel (Yescarta®), brexucabtagene autoleucel (Tecartus®), lisocabtagene maraleucel (Breyanzi®), idecabtagene vicleucel (Abecma®), and relmacabtagene (Relma-cel®). Besides, some of the CAR-T therapies are being introduced at national levels, such as ciltacabtagene autoleucel (Carvykti®) approved by the National Medical Products Administration in China (Johnson and Abramson 2022) or ARI-0001 (CART19-BE-01) authorized by the Spanish Agency of Medicines and Medical Devices under the “hospital exemption” approval pathway (Trias et al. 2022).

In a traditional approach, generation of CAR-T cells for the therapy is associated with the collection of patient’s T lymphocytes and their in vitro modification, which results in a surface expression of CAR that enables the cells to specifically recognize and attack target cells. Next, CAR-T cells are expanded in vitro and injected back into the patient’s bloodstream (Jogalekar et al. 2022). The complexity of a classical protocol for preparing CAR-T cells is one of the most pronounced disadvantages of this therapy, as it makes the preparation time-consuming (about three weeks) and is responsible for significantly high costs of this treatment (up to $500,000 per patient) (Sterner and Sterner 2021). Those problems would be solved by a successful protocol to produce CAR-T cells in situ, i.e., within the patient’s body. Therefore, elaboration of such protocol(s) is one of the most pressing needs for the CAR-based therapies. This review aims to summarize the current state-of-the-art and future perspectives for the in situ produced CAR-T cells.

## CAR Structure

The basic structure of CAR is composed of three regions: ectodomain, transmembrane domain, and endodomain, which can be further divided into functional domains. Variations in each component of the receptor enable adjusting the antitumor activity of the resultant CAR-T cell, as well as improving their efficiency and safety (Jayaraman et al. 2020).

The extracellular part of the CAR (endodomain), also called antigen recognition and binding domain, is responsible for recognizing target antigens in a non-major histocompatibility complex (MHC)-dependent manner with high affinity and directing the cytotoxic function of T cells to kill a specific type of cell (Jayaraman et al. 2020). Classically, the antigen-binding function has been performed by single chain variable fragments (scFv) formed by combining variable heavy and variable light chains of monoclonal antibodies, linked by a flexible linker like (Gly4Ser)3, which ensures solubility and flexibility of the scFv (Bailey and Maus 2019). Traditionally, scFv sequences target extracellular antigens present on cancer cells. However, CARs with scFvs targeting soluble ligands in the tumor microenvironment (TME) have also been engineered (Dwivedi et al. 2019). Nanobodies, also known as VHH, are another antibody-based antigen-binding domains used in CARs. Those variable domains of the heavy chain only antibodies have similar specificity and affinity to conventional antibodies (Arbabi-Ghahroudi 2017; Chang et al. 2018). Due to their smaller size, they are more suited for reaching less-accessible epitopes on solid tumors than scFv (Hassani et al. 2019; Jamnani et al. 2014). Furthermore, they are characterized by low immunogenicity, high solubility, stability, and tissue penetration, which makes them a feasible alternative to scFv in CARs (Chang et al. 2018). Other options for the antigen recognition and binding domain, such as the use of natural ligands or artificial connectors are also possible.

The other part of the CAR present in the external region of the cell is the hinge. Being responsible for the linkage of the extracellular and transmembrane domain, it provides the antigen recognition domain with adequate space and flexibility to form a connection between the T-cell and the target cell. Sequences from constant regions of immunoglobulins (such as IgG1 or IgG4), CD8α or CD28 proteins have been playing the role of hinge regions in CARs (Guedan et al. 2019; Kochenderfer et al. 2009; Milone et al. 2009).

The transmembrane domain is the region of the CAR that links extracellular antigen recognizing part of the receptor with intracellular signaling domains while anchoring the CAR to the membrane and stabilizing it (Fujiwara et al. 2020). Usually, it is composed of type I proteins, such as CD28, CD8α, CD4, or CD3ζ. Each transmembrane domain has unique properties allowing fine-tuning receptor’s properties. For example, CAR-T cells with a CD8α transmembrane domain release less tumor necrosis factor α and interferon (IFN)-γ, making them less susceptible to activation-induced cell death (Alabanza et al. 2017). CD3ζ transmembrane domain, in turn, interacts with endogenous T-cell receptor (TCR) facilitating T-cell activation (Bridgeman et al. 2010). Moreover, CARs with CD28 transmembrane domain are more sensitive to low antigen density compared to CARs with CD8α transmembrane domain (Majzner et al. 2020).

CAR intracellular signaling domain is the functional part of the receptor typically comprising of an activation domain and one or multiple co-stimulatory domains (Guedan et al. 2019). Activation, in the majority of CARs, is mediated by ζ-chain of CD3 cluster (CD3ζ)-derived immunoreceptor tyrosine-based activation motifs (ITAMs). ITAMs are phosphorylation sites that recruit ZAP70 essential for signaling cascades. However, it was found that signal mediated by CD3ζ-derived ITAMs is insufficient for satisfactory T-cell activation and persistence in vivo (Bridgeman et al. 2010). Therefore, co-stimulatory domains have been added in later designs (Savoldo et al. 2011). Most commonly used co-stimulatory domains are derived from 4-IBB (CD137) and/or CD28. T cells with receptors containing 4-1BB domain differentiate into central memory T cells and are characterized by enhanced mitochondrial oxidative phosphorylation, persistence, and rate of proliferation. CARs with CD28 domain differentiate into effector memory T cells increasing their reliance on aerobic glycolysis (Kawalekar et al. 2016). Alternative co-stimulatory domains have also been explored, including inducible T-cell co-stimulator, OX40 receptor (CD134), CD27, MYD88, CD40, and killer cell immunoglobulin-like receptor 2DS2 (Honikel and Olejniczak 2022).

## CARs Generations

**Fig. 1:**
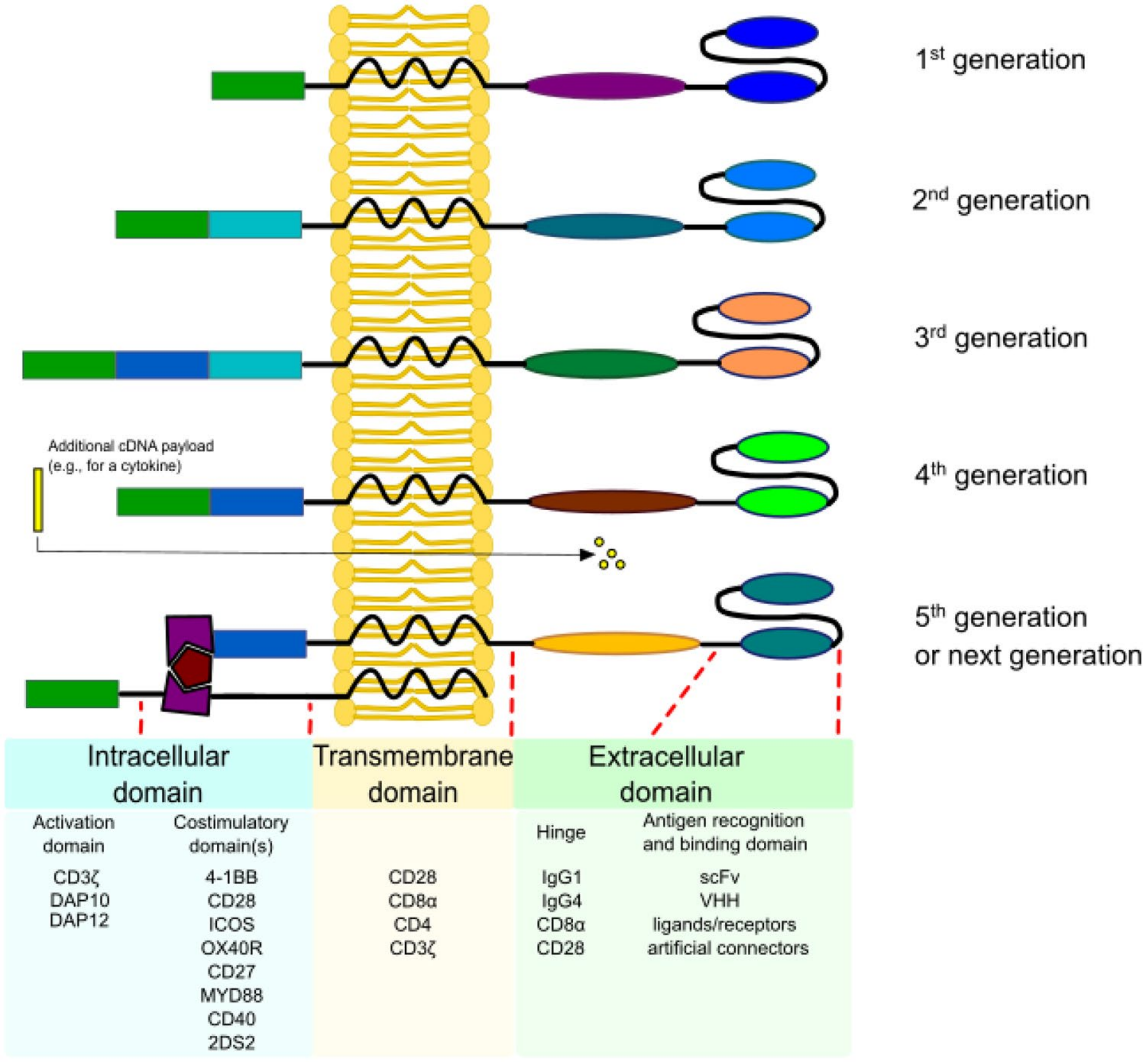
Evolution of CAR structure throughout different generations. First generation of CARs contains only antigen recognition domains and CD3ζ activation domain. Second generation of CARs additionally comprises a co-stimulatory domain (such as 4-1BB or CD28). In the third generation, two co-stimulatory domains are included. T cells expressing fourth generation CAR (sometimes called TRUCKs) contain an additional cDNA cassette, e.g., encoding an immunomodulator. Fifth generation of CARs is a group of next gen receptors, such as ON-switch CARs (on the figure above), the universal CAR-T cells, etc., all aiming at improving safety and efficiency of immunotherapies

CARs were first designed in 1989 by Gross et al. as a way to utilize antibody-like specificity to direct T-cell effector functions in a non-MHC restricted manner (Gross et al. 1989). Throughout the years, CARs underwent many modifications, which were intended to improve safety of the therapy by reducing toxicity and non-specific antigen recognition, to increase the efficiency by stimulating proliferation, activation, and generation of memory phenotype in CAR-T cells, and also to provide immunomodulation for optimal actions of CAR-T cells (Fig. 1).

First generation of CARs combined with scFv, as an antigen-binding domain, with CD3ζ, as an intracellular signaling activation domain (Eshhar et al. 1993). The major shortcoming of first-generation CARs was lack of co-stimulatory domains, which resulted in inability of cells to produce sufficient levels of interleukin (IL)-2 leading to low cytotoxicity and proliferation levels (Brocker 2000; Kershaw et al. 2006; Lamers et al. 2006; Park et al. 2007). Therefore, in the second-generation CARs, a co-stimulatory molecule (such as CD28 or 4-1BB) was added, which resulted in enhanced T-cell proliferation and in vivo survival (Savoldo et al. 2011).

To further enhance the efficiency of CAR-T cells, two co-stimulatory domains were included in the third-generation CAR constructs. The goal of this modification was to combine cytotoxic capabilities of CARs equipped with CD28 with the persistence granted by the 4-1BB domain (Weinkove et al. 2019). CAR constructs with two co-stimulatory molecules were characterized by the highest rate of tumor eradication, complete remission in the long term, and survival at lower doses of CAR-T cells (Schubert et al. 2019; Zhao et al. 2015).

The fourth CAR generation is sometimes referred to as TRUCK (T cells redirected for antigen-unrestricted cytokine-initiated killing). It combines cytolytic capacities of T cells with immunomodulating effects of cytokines. TRUCKs are able to recruit other immune cells via a CAR-inducible transgenic product to assist in antitumor response and overcoming the immunosuppressive TME (Chmielewski et al. 2014). To generate TRUCKs, T cells are transduced with an inducible cassette with an immunomodulator, such as IL-12, IL-18, and IL-23, expression of which is dependent on nuclear factor of activated T cells (Chmielewski et al. 2014; Zhang et al. 2011).

The next, fifth generation of CARs, equipped with various molecular mechanisms, is a response to the growing demand for safer and more effective adoptive immunotherapy (Tomasik et al. 2022). An example of such an approach is adding the PD-1/CD28 chimeric switch-receptor, which increases the efficacy of therapy (Liu et al. 2021a). Other switchable CAR-T cells were generated to prevent therapy adverse effects, including cytokine release syndrome (CRS). These CARs are equipped with a safety switch—an easily targetable surface molecule, which enables creation of an immunological synapse with tumor cells, and can be easily depleted by pharmaceutical agents (Moghanloo et al. 2021).

Moreover, to overcome the difficulties related to conventional manufacturing the CAR-T cells from patient-derived autologous T cells, the universal CAR-T cells were generated. During their production, both TCR and MHC are being removed from donor cells by CRISPR/Cas9- or transcription activator-like effector nuclease-based techniques (Rafiq et al. 2020). This can provide an off-the-shelf approach in CAR-T-based therapies. However, due to imperfection of gene-edition techniques, a potential risk of mutagenesis exists. Thus, a more desired solution for this problem may be generation of CAR-T cells directly in patient’s body, i.e., in situ.

## Advantages of In Situ Generation of CAR-T Cells

**Fig. 2:**
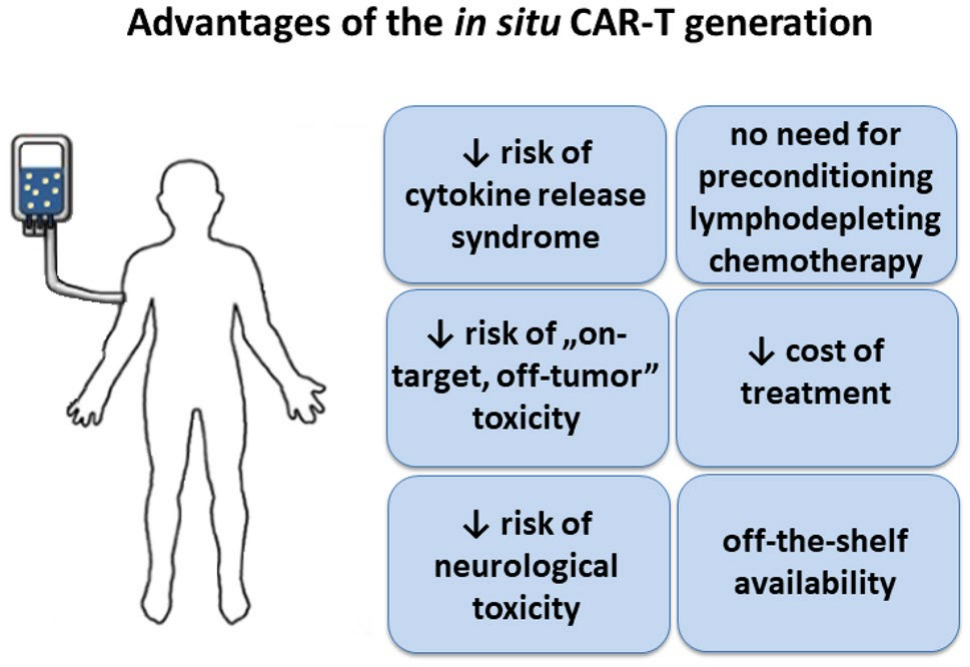
Most prominent advantages of the in situ CAR-T generation

As mentioned above, CAR-T therapy with an ex vivo-generated cells has multiple barriers associated with complicated and expensive manufacturing, as well as high risk of toxicity (Jogalekar et al. 2022; Neelapu 2019). Therefore, the in situ induced CAR-T cells could be a potential solution to these issues. The undoubted advantage of the in situ CAR-T generation is the facilitation and the possibility of standardization of production, which significantly reduces the cost of treatment. Moreover, it was demonstrated that in situ CAR-T induction is associated with reduced risk of the most common toxicities associated with CAR-T therapy, such as CRS, immune effector cell-associated neurotoxicity syndrome, and “on-target, off-tumor” toxicity (Xin et al. 2022). Other important features associated with the in situ manufactured CAR-T cells are: no need for preconditioning lympho-depleting chemotherapy before CAR-T cells infusion and lower risk of resistance to CAR-T cell treatment (Fig. 2).

## Advances in the In Situ Generation of CAR-T Cells and Their Application

In currently approved therapies, CAR-T cells are produced ex vivo mainly in the lentiviral systems or by non-viral delivery platforms.

### *Lentiviral Systems for *In Situ* CAR-T Generation*

This platform allows stable integration of large cDNA sequences of transgenes into the transduced cells (Frimpong and Spector 2000). However, the manufacturing process is multistep and complicated, including collection of T cells from patient’s material, transduction, expansion of the modified T cells, and infusion of generated CAR-T cells into patient’s bloodstream (Jogalekar et al. 2022). As mentioned above, this technology is costly, time-consuming, and complex (Sterner and Sterner 2021). Therefore, some new approaches have been explored, including in situ virus-mediated CAR-T generation. Indeed, several studies have been conducted to evaluate whether the injection of surface-engineered viral vectors expressing specific CARs can reduce the number of tumor cells in experimental models (Frank and Buchholz 2018). The principle behind this approach is pseudotyping of viral particles for precise transduction of the vectors into specific type of immune effector cells. For instance, Pfeiffer et al. (2018) demonstrated that CD19-CAR-T cells generated in vivo by the lentiviral vector CD8-LV in humanized mice specifically targeted human CD8^+^ cells and were able to eliminate CD19^+^ B cells and Raji cells. In another study, the same group analyzed if such CAR-T cells are able to entirely eliminate the luciferase-encoding CD19^+^ Nalm-6 tumor cells from bone marrow and spleen of T cells transplanted NSG mice (Agarwal et al. 2019). It was revealed that two weeks after CD8-LV vector injection, the complete tumor remission was observed in 50% of mice. In another 37.5% of animals the remission was observed at day 17. Moreover, it was found that 5–12% of human CD3^+^CD8^+^ cells isolated from mouse bone marrow, spleen, and blood were CAR-positive, and the CD8^–^ cells were CAR-negative. Next, the authors analyzed CAR expression in mouse NK cells, as well as natural killer T (NKT) cells. CAR^+^ NK and NKT cells were observed in bone marrow and spleen on day 14 and 18, which suggests a non-specific action of the CD8-LV vector.

To enhance specificity of the lentiviral vector for human T cells, Huckaby et al. (2021) generated Sindbis lentiviral vector with a bispecific antibody binder. It was demonstrated that a single dose of this vector generated CAR-T cells from circulating T lymphocytes in a humanized NSG mice injected with FFLuc BV-173 malignant B cells. These CAR-T cells suppressed CD19^+^ tumor cell growth and prolonged the overall survival time of mice.

In a study of Nawaz et al. (2021), CAR-encoding adeno-associated viral vector was used, and it confirmed a single vector infusion into animals of humanized NOD. Cg-Prkdcscid Il2rgem26/Nju tumor mouse model of human T-cell leukemia reprogrammed T cells to express CAR. In addition, the authors observed tumor reduction at day 10. However, the use of this vector caused the appearance of CAR^+^ NK cells and CAR^+^ B cells.

The aim of a study conducted by Zhou et al. (2015) was to increase the viral vector cell specificity. This group targeted lentivirus by pseudotyping with modified envelope proteins. CD4-targeting was achieved by fusion of envelope proteins of measles virus with CD4-specific designed ankyrin repeat protein, while CD8 selectivity was accomplished with modified envelope proteins of Nipah virus fused to a CD8-specific single chain variable fragment*. *In vivo-generated CAR-T cells contributed to tumor regression in mouse models.

Despite the proof-of-concept studies described above, there are still numerous obstacles to overcome before the targeted lentiviral CAR-T will be approved for treatment in humans. Among others, foreign antigens on the viral envelope, recognized and phagocytosed by antigen presenting cells, might trigger innate immune responses against viral particles, which would limit the vector stability (Breckpot et al. 2010). Furthermore, lentiviral platform enables large DNA inserts to integrate favoring sites near active genes, which compels a precautionary approach in treatment (Braun et al. 2014; Hacein-Bey-Abina et al. 2003; Lana and Strauss 2020). In addition, production of sufficient quantities of biologically active virions in large scale poses an issue. The targeted lentiviral vectors have a 100-fold lower titer than VSV-G lentivirus (Zhou et al. 2012). Lower efficiency of lentiviruses generation raises costs of treatment and hinders scaling-up of whole process.

### *Non-viral Delivery Systems for *In Situ* CAR-T Generation*

As an alternative to viral platforms, numerous non-viral delivery systems are being developed. The vectors can be either regular mammalian expression plasmids (Zhang et al. 2018), transposon-based systems (Lock et al. 2022) or mRNA (Foster et al. 2019) (for examples of gene transfer platforms see Table 1).

**Table 1:** Examples of gene transfer platforms and their short characteristic

	Advantages	Disadvantages	Example	Characteristic	Disadvantages
Viruses	- High efficiency of transfection - Enables long-lasting modification	- Limited insert size - Difficult production in large scale - Risk of generation of replication competent viruses - Risk of in vivo recombination with other viruses’ sequences - Risk of immunogenicity connected with humoral and cellular immune response	Gammaretroviruses	- Integrate near transcription start sites - Can only infect dividing cells	- Higher risk of oncogenesis
			Lentiviruses	- Integrate near transcriptionally active regions - Infect both dividing and non-dividing cells - More complex production	- Higher likelihood of disturbing gene expression
Transposons	- Easy production of large quantities of plasmids - 5–10 times lower costs than in viral process - Higher insert size - Can modify dividing and non-dividing cells - Low immunogenicity	- Low risk of an interaction with endogenous human DNA sequences	Sleeping Beauty	- Optimal cargo is under 6 kb, but it’s possible to increase it up to 11 kb or even 100 kb by combination with BAC	- Significant toxicity caused by electroporation - Possible horizontal dissemination of antibiotic resistance gene into pathogenic bacteria
			PiggyBac	- Provides precise excision from an insertion site and restores pivotal sequence with no requirement of DNA synthesis - Optimal cargo capacity is up to 14 kb - Higher transposition activity than SB - Using multicistronic cassettes enables for multiple transgene delivery	- Significant preference for integration near TSSs, CpG islands and DNase I hypersensitive sites which increases risk of gene dysregulation -Higher probability to associate with oncogenes -The presence of PGBD5 might be a source of gene dysregulation
mRNA	- No risk of genotoxicity - Flexible system	- Transient transfection - For prolonged expression only non-proliferating cells could be used - Low efficiency	Physical delivery	- Low immunogenicity	- Cannot be used on internal organs
			Chemical delivery	- Flexible composition enables for adjustment of formulation to specific target	- Due to route of administration might modify also untargeted cells

The non-viral techniques can be roughly divided into physical and chemical methods. Instances of physical methods are the electroporation, needle injection, laser irradiation, and gene guns. Chemical methods include nanoparticles (lipid, polymeric, golden, and silica), quantum dots, carbon nanotubes, exosomes, ferritin, and cell membranes (Xin et al. 2022). Generally, physical techniques have low immunogenicity; however, they cannot be used in internal organs. In turn, characteristics and potential for clinical application of chemical methods vary, depending on particles used in formulation. Out of these, lipid nanoparticles (LNPs) have recently successfully entered the clinic for the delivery therapeutics (Algarni et al. 2022; Mukai et al. 2022). LNPs are composed of cholesterol and helper lipids ensuring the integrity of the particles, a PEGylated lipid maintaining colloidal stability and restricting aggregation in reticuloendothelial system, and an ionizable amine-containing lipid, which is crucial for optimal formulation due to its role in complexation of nucleic acid (Eygeris et al. 2022). It was confirmed that therapeutic mRNA-loaded LNPs injected intravenously are endocytosed by various types of cells, mainly hepatocytes (Pardi et al. 2015). During the endosome escape, mRNA is released into the cytoplasm, where it is translated into proteins (Akinc et al. 2010; Pardi and Weissman 2017). Due to numerous modifications in formula, LNPs can deliver either DNA or RNA. For instance, packing nucleic acid spherically around a nanoparticle template increases engagement of scavenger receptor, which results in higher accumulation in cells (Choi et al. 2013). However, platforms based on RNA are more successful so far, with Comirnaty® SARS-CoV-2 mRNA vaccine by BioNTech/Pfizer, mRNA-1273 SARS-CoV-2 mRNA vaccine by Moderna and Onpattro transthyretin siRNA for hereditary amyloidosis by Alnylam (Milane and Amiji 2021). Moderna also has several mRNA vaccine candidates in clinical trials: mRNA-4157, a personalized cancer vaccine in phase 2 clinical trials for the treatment of melanoma (ClinicalTrial.gov identifier: NCT03897881), and mRNA-5671, a KRAS vaccine in phase 1 clinical trials for the treatment of pancreatic, colorectal and non-small cell lung cancers (NCT03948763). LNP architecture enables also chemotherapeutics accumulation. In this space, Moderna has two formulations in Phase 1 clinical trials: mRNA-2752 encapsulating mRNA encoding OX40L, IL-23, and IL-36 (NCT03739931, NCT02872025) and MEDI1191 encapsulating mRNA for IL-12 (NCT03946800) (Schallon et al. 2012).

Importantly, LNPs platform enables directing particles by both changing the formulation (passive targeting) of LNPs and adding target-specific ligands (active targeting). An example of passive targeting, achieved by Nakamura et al. (2020), is increasing the quantity of DMG-PEG2000 which resulted in decreased size of LNPs with higher chance of uptake by dendritic cells in lymph nodes. The same group also enhanced cellular uptake by creating negatively charged LNPs with CHEMS at ~ 20 moll%. The other method used to obtain higher LNPs selectivity was replacing PEG-lipids with 3% Tween 20 (Zukancic et al. 2020), differing ratios of DODAP and DOPE lipids (Kimura et al. 2021), and changing ionizable lipid DLin-MC3-DMA to DLin-KC2-DMA (Dilliard et al. 2021). There are also new lipids added to the formula by dissolution at different molar ratios in ethanol or THL, termed as selective organ targeting lipids (Dilliard et al. 2021) which increase liver, spleen, and lung targeting (Álvarez-Benedicto et al. 2022; Cheng et al. 2020; Lee et al. 2021; Liu et al. 2021b). Introducing target-specific ligands was accomplished by adding DSPE-PEG at 12.5–25 mol% of total PEG to the formula (Li et al. 2020). This modification enables to engraft specific antibody chemically by amidation for αCD34 antibody (Kedmi et al. 2018), a Diels–Alder reaction for Fab-C4 (Li et al. 2020), or conjugating anti-CD4 antibody to thiol-maleimide (Ramishetti et al. 2015; Tombácz et al. 2021) and PECAM-1-specific monoclonal antibody to DSPE-PEG-maleimide (Parhiz et al. 2018). Ligands can also be attached to a cholesterol, for example, α-mannose containing an aminopropyl succinate spacer via an amide bond, in order to target dendritic cells (Goswami et al. 2019).

A few studies described the in situ generation of CAR-T cells using nanoparticles (NPs) as potentially ideal reagents which could be commercially manufactured, stored, and delivered.

Smith et al. (2017) designed biodegradable poly-(β-amino ester)-based NPs and encapsulated into them two plasmids encoding the leukemia-specific 194-1BBz CAR (a fusion receptor specific for the extracellular domain of CD19) and hyperactive iPB7 transposase. The group demonstrated that NPs selectively bind CD3^+^ lymphocytes, and that these cells after the transfection were functional, non-toxic, and underwent proliferation. Moreover, it was found that nanocarriers were able to reduce tumors in albino C57BL/6 mice.

The same poly-(β-amino ester)-based NPs were used to deliver in vitro-transcribed mRNA encoding CAR or TCR for reprograming of T cells (Parayath et al. 2020). These NPs transfected human CD8^+^ T cells and were able to reprogram circulating T cells to recognize leukemia in immunodeficient NOD.Cg-Prkdcscid Il2rgtm1Wjl/SzJ (NSG) mice. Additionally, NPs showed the antitumor activity in NSG mice subcutaneously injected with LNCaP C42 prostate carcinoma cells, as well as in HBV-induced hepatocellular carcinoma.

Kang et al. (2021) developed another method of encapsulating piggyBac vector coding anti-anaplastic lymphoma kinase CAR (ALK-CAR) into a mannose-conjugated polyethylenimine (MPEI) nanoparticle, and generating ALK-CAR-M in vivo*.* PEI, as a well-studied cationic polymer, is commonly used as a cell transfection agent. However, in this study, it was conjugated with mannose, a ligand for a mannose receptor overexpressed in macrophages, which allowed for a more specific targeting of nanoparticles. Interestingly, scientists decided to also deliver gene coding IFN-γ to modified in situ CAR-M in order to polarize cells from pro-tumoral M2 into an anti-tumoral M1 phenotype. The MPEI/pCAR-IFN-γ-transfected macrophages indeed have changed their phenotype to M1 in vitro, but also delayed the tumor growth in Neuro-2a-bearing mice after both intra-tumoral and intraperitoneal injection. The MPEI/pCAR-IFN-γ injection led to an increase in activated CD8^+^ T-cell and a decrease in CD4^+^CD25^+^FoxpP3^+^ regulatory T-cell populations in the tumor. With 7.6–13% of CD11b^+^ CAR-IFN-γ-positive macrophages in the tumor 16 days after the injection, the results indicate that MPEI-mediated modification enables generating functional CAR-M in situ.

An interesting study followed the proof-of-concept observation of Aghajanian et al. (2019) in a mouse model of angiotensin II/phenylephrine (AngII/PE)-induced cardiac injury and fibrosis, which showed efficacy in anti-fibroblast activation protein (FAP) CAR-T cells in elimination of cardiac fibroblasts and alleviation of the fibrosis progression. Subsequently, Rurik et al. (2022) generated antifibrotic CAR-T cells in vivo by LNP-mediated delivery and evaluated their effects in this mouse model of heart injury. To this end, LNPs coated with antibodies against CD5 were used to deliver mRNA construct and selectively reprogram T cells into FAP-targeting CAR-T cells. It was observed that after 48 h from LNPs injection, there were 17.5–24.7% of FAP-CAR-positive T cells and that the expression of FAP-CAR was transient and vanished in splenic T cells after one week post injection. In vivo LNP-generated CAR-T cells killed FAP-expressing cells in vitro, and were capable of trogocytosis. The group also evaluated if in vivo-generated CAR-T cells were able to improve cardiac function in mice. Indeed, it was demonstrated that 14 days after the single injection, left ventricular end diastolic and end systolic volumes were normalized. Histologic analysis also revealed a significant improvement in the overall burden of extracellular matrix. Those results provide a proof of concept that LNPs can deliver mRNA specifically and modify T cells in situ to produce functional CAR-T.

Rapid development of LNP-mRNA delivery platform has brought new perspectives to the design of modern therapeutics. With its transient nature, LNPs enable precise dosing, reduced toxicity, and make it possible to make numerous modifications in formula, reducing random uptake and increasing targeting of the therapeutics. Therefore, this modern delivery platform will become the future of design of cellular therapies.

Main studies on in situ-generated CAR cells are summarized in Table 2.

**Table 2:** Main studies on in situ generated CAR cells

Gene transfer platform	Relevant results	References
Viral		
Lentiviral vector CD8-LV	CD19-CAR-T cells generated in humanized mice specifically targeted human CD8^+^ cells and eliminated CD19^+^ B cells and Raji cells	Pfeiffer et al. (2018)
Lentiviral vector CD8-LV	CD19-CAR-T cells generated in humanized mice eliminated luciferase-encoding CD19^+^ Nalm-6 tumor cells from bone marrow and spleen of T cells transplanted NSG mice CAR^+^ NK and NKT cells were observed, which suggests a non-specific action of the vector	Agarwal et al. (2019)
Sindbis lentiviral vector with a bispecific antibody binder	A single dose of vector generated CAR^+^ T cells in a humanized mice injected with FFLuc BV-173 malignant B cells. These CAR-T cells suppressed CD19^+^ tumor cell growth and prolonged the overall survival time	Huckaby et al. (2021)
Adeno-associated viral vector	A single vector infusion into humanized mouse model of human T-cell leukemia reprogrammed T cells to express CAR. These CAR-T cells suppressed tumor growth CAR^+^ NK cells and CAR^+^ B cells were observed, which suggests a non-specific action of the vector	Nawaz et al. (2021)
Lentiviral vectors CD4-LV and CD8-LV	CD4-LV and CD8-LV vectors infusion selectively transduced human CD4^+^ and CD8^+^ T cells. These CAR-T cells contributed to tumor regression in mouse models	Zhou et al. (2012, 2015)
Non-viral		
Poly (β-amino ester)-based NPs	NPs with encapsulated plasmids coding leukemia-specific 194-1BBz CAR and hyperactive iPB7 transposase selectively bound CD3^+^ lymphocytes. Cells after the transfection were functional, non-toxic, and underwent proliferation Nanoparticles reduced tumors in albino C57BL/6 mice	Smith et al. (2017)
Poly (β-amino ester)-based NPs	NPs delivering mRNA encoding CAR or TCR transfected human CD8^+^ T cells and reprogrammed circulating T cells to recognize leukemia in immunodeficient mice. NPs showed the antitumor activity in mice subcutaneously injected with LNCaP C42 prostate carcinoma cells and in HBV-induced HCC	Parayath et al. (2020)
Mannose-conjugated polyethylenimine NPs	NPs with encapsulated piggyBac vector coding anti-ALK CAR and generated ALK-CAR macrophages (M). To polarize cells from M2 into M1 phenotype, a gene coding IFN-γ was delivered to modified CAR-M. Transfected macrophages changed their phenotype to M1 and delayed the tumor growth in tumor-bearing mice NPs injection increased the number of activated CD8^+^ T cells and decreased CD4^+^CD25^+^FoxpP3^+^ regulatory T cells in the tumor	Kang et al. (2021)
LNPs	CD5-coated LNPs delivering mRNA encoding FAP selectively reprogrammed T cells into FAP-targeting CAR-T cells. CAR-T cells killed FAP-expressing cells in vitro and were capable of trogocytosis. 14 days after LNPs injection, left ventricular end diastolic and end systolic volumes were normalized in a mouse model of heart injury	Aghajanian et al. (2019); Rurik et al. (2022)

## Conclusions and New Potential Directions of Application of In Situ Generated CAR-T/NK/M Cells

Efficient, rapid, and safe generation of CAR-bearing immune effector cells directly within the patient’s body is one of the ‘Holy Grails’ in the CAR-related research. There are numerous potential advantages of such an approach over traditional protocols for ex vivo generation of CAR-T/NK/M cells (see Fig. 2). It is quite unlikely, however, to elaborate a single type of universal protocol for in situ production of CAR-bearing cells as various diseases would need specialized approaches. Generally, for cancer-targeted CARs, one would need a long-lasting persistence of memory-type cells to ensure the immunosurveillance over the residual disease and to prevent the recurrence of cancer. Thus, methods of generating stable expression of CARs in long-lasting cells, e.g., by surface-engineered viruses or transposon-employing systems, are of greater value for oncological research. Such approaches would need very high precision of vector targeting, to avoid stable modification of bystander cells with all potential consequences of such. Conversely, in autoimmune-, allergy-, amyloidosis-, or fibrosis-related illnesses, the hit-and-run strategies may be more suitable, in order to decrease the risk of late toxicities. Therefore, transient expression methods, e.g., mRNA-based, could be of better use thereby. However, a long-lasting expression of CARs in regulatory T cells might be desired as well, at least in theory. An open question is, how the in situ CAR delivery techniques could be employed in other areas of medicine, such as infectious diseases or transplantology. It must be also noted that at present stage of development, the in situ methods for CAR delivery proved useful for simple single-CAR expression systems. The challenge lies in making them more suitable for sophisticated conditional-activity or sensor-effector types of strategies, which may be of importance in case of CAR targets present ubiquitously in vital organs under normal conditions (Bajor et al. 2022). Altogether, given the encouraging results of the abovementioned proof-of-concept studies in animal models, one should expect prospective translational development and validation in practical medicine of strategies for in situ generation of CAR-bearing immune effector cells. For now, many questions remain before in situ CAR-T therapy can reach the clinic.

## Data Availability

Data sharing is not applicable to this article as no datasets were generated or analyzed during the current study.
