# Correction to: In Situ Programming of CAR-T Cells: A Pressing Need in Modern Immunotherapy

**DOI:** 10.1007/s00005-023-00687-8

**Published:** 2023-09-02

**Authors:** Marta Śledź, Alicja Wojciechowska, Radosław Zagożdżon, Beata Kaleta

**Affiliations:** Department of Clinical Immunology, Medical University of Warsaw, Warsaw, Poland

**Correction to: Archivum Immunologiae et Therapiae Experimentalis (2023) 71:18** 10.1007/s00005-023-00683-y

Authors would like to correct the incorrect journal name in the Consent for Publication section. The correct version is updated here.

Consent for Publication: All authors have read the manuscript and agreed to give their consent for the publication of information in the Archivum Immunologiae et Therapiae Experimentalis.

The original article has been corrected.

